# Patterns of tobacco use in the United Arab Emirates Healthy Future (UAEHFS) pilot study

**DOI:** 10.1371/journal.pone.0198119

**Published:** 2018-05-30

**Authors:** Mohammed Al-Houqani, Andrea Leinberger-Jabari, Abdullah Al Naeemi, Abdullah Al Junaibi, Eiman Al Zaabi, Naima Oumeziane, Marina Kazim, Fatima Al Maskari, Ayesha Al Dhaheri, Leila Abdel Wareth, Wael Al Mahmeed, Habiba Alsafar, Fatme Al Anouti, Abdishakur Abdulle, Claire K. Inman, Aisha Al Hamiz, Muna Haji, Jiyoung Ahn, Tomas Kirchhoff, Richard B. Hayes, Ravichandran Ramasamy, Ann Marie Schmidt, Omar El Shahawy, Michael Weitzman, Raghib Ali, Scott Sherman

**Affiliations:** 1 College of Medicine and Health Sciences, United Arab Emirates University, Al Ain, United Arab Emirates; 2 Public Health Research Center, New York University Abu Dhabi, Abu Dhabi, United Arab Emirates; 3 Zayed Military Hospital, Abu Dhabi, United Arab Emirates; 4 Department of Pathology, Sheikh Khalifa Medical Center, Abu Dhabi, United Arab Emirates; 5 Pathology & Laboratory Medicine Institute, Cleveland Clinic Abu Dhabi, United Arab Emirates; 6 Heart and Vascular Institute, Cleveland Clinic Abu Dhabi, Abu Dhabi, United Arab Emirates; 7 Department of Biomedical Engineering, Khalifa University, Abu Dhabi, United Arab Emirates; 8 College of Natural and Health Sciences, Zayed University, Abu Dhabi, United Arab Emirates; 9 Department of Population Health, NYU School of Medicine, New York, New York, United States of America; 10 Permutter Cancer Center, New York University, New York, New York, United States of America; University of Cape Town Lung Institute, SOUTH AFRICA

## Abstract

**Introduction:**

Self-reported tobacco use in the United Arab Emirates is among the highest in the region. Use of tobacco products other than cigarettes is widespread, but little is known about specific behavior use patterns. There have been no studies that have biochemically verified smoking status.

**Methods:**

The UAE Healthy Future Study (UAEHFS) seeks to understand the causes of non-communicable diseases through a 20,000-person cohort study. During the study pilot, 517 Emirati nationals were recruited to complete a questionnaire, provide clinical measurements and biological samples. Complete smoking data were available for 428 participants. Validation of smoking status via cotinine testing was conducted based on complete questionnaire data and matching urine samples for 399 participants, using a cut-off of 200ng/ml to indicate active smoking status.

**Results:**

Self-reported tobacco use was 36% among men and 3% among women in the sample. However, biochemical verification of smoking status revealed that 42% men and 9% of women were positive for cotinine indicating possible recent tobacco use. Dual and poly-use of tobacco products was fairly common with 32% and 6% of the sample reporting respectively.

**Conclusions:**

This is the first study in the region to biochemically verify tobacco use self-report data. Tobacco use in this study population was found to be higher than previously thought, especially among women. Misclassification of smoking status was more common than expected. Poly-tobacco use was also very common. Additional studies are needed to understand tobacco use behaviors and the extent to which people may be exposed to passive tobacco smoke.

**Implications:**

This study is the first in the region to biochemically verify self-reported smoking status.

## Introduction

The WHO estimates tobacco use in the Eastern Mediterranean Region (EMR) to be nearly 25%, compared to worldwide prevalence of 22.7% (age-standardized prevalence).[1] Additional studies have examined tobacco use in the region that are country-specific and have typically relied on cross-sectional data.[2–9] In the EMR, alternative forms of tobacco use are common. Waterpipe, also known as “shisha”, “hookah” or “narghile”, has been used for many years and is gaining in popularity in the region and elsewhere. [4,8,10–13] Use of “dokha” tobacco, another alternative form of tobacco consisting of tobacco mixed with herbs and smoked with a “midwakh” pipe is also common to the region.[2,6,14,15] However, there are limited data on non-cigarette tobacco product use on a country-specific level within the EMR, and specifically within the Gulf Cooperation Council (GCC).

The United Arab Emirates (UAE) is an emerging country that is comprised of seven emirates, Abu Dhabi being the largest with an estimated population of 2.7 million. Access to primary care services is similar to access in the US and other Western nations.[16] Despite this relatively good access to primary care, Abu Dhabi has high rates of chronic disease. In 2015, cardiovascular diseases accounted for nearly 35% of all deaths.[16] Cancer, is the second highest cause of death, and cancer of the lung, bronchus and trachea accounted for 14% of all cancer deaths in the emirate.[16] Tobacco use is a major contributing factor to the high rate of non-communicable diseases in the UAE.

In the UAE, tobacco use amongst UAE nationals is reported to be approximately 24% in men and much lower in women (0.8%).[7,15] Tobacco use has been reported to be highest in young men between the ages of 30–39 (28%), Arab expatriate men (31.4%) and non-Arab expatriate women (10.7%).[6,15] Among UAE Nationals, smoking rates for men were highest among 20–29 year olds (29%) and 30–39 year olds (32%), and rates were very low among UAE women (0.7%).[7] However, all of the studies to date in the UAE have relied upon self-reported smoking status without biochemical verification. There is mixed evidence about the accuracy of self-reported smoking estimates in a population.[17–21] To gain a more accurate estimate of prevalence of smoking in the UAE we biochemically verified responses to smoking behavior queries in a population-based survey.

## Materials and methods

We are conducting the first prospective cohort study of the Emirati population aimed at understanding the factors that contribute to cardiovascular disease, diabetes and obesity. The UAE Healthy Future Study (UAEHFS) seeks to recruit 20,000 Emirati adults between the ages of 18–40 to understand these health conditions. Participants are eligible if they are older than 18 years, an Emirati national, can speak and read in Arabic or English, and able to provide consent. Non-Emiratis and pregnant women were excluded from the study.

We conducted an initial pilot study to determine the feasibility of the larger prospective cohort. Data were collected from 517 participants who met the study inclusion criteria, via questionnaire, clinical measurements and biological samples for laboratory analysis. Emirati nationals were recruited from 2 health care centers located in the Emirate of Abu Dhabi. Participants completed an online survey that included questions on demographics, individual and family health history, diet and physical activity, and smoking.

### Self-reported smoking status

The questions in the smoking section were adapted from the UK Biobank Study, and asked about current and past tobacco use.[22] Self-reported smoking was assess by three questions: “Do you smoke cigarettes now?”, “Do you smoke Midwakh/Dokha/pipe now?”, and “Do you smoke shisha/Waterpipe now?”

### Cotinine validation

Urine samples were collected from participants to test for the presence of cotinine, a metabolite of nicotine. Urine samples were collected at the recruitment sites in individual containers, couriered to the lab and frozen until the time of analysis. Urine cotinine tests can produce an accurate estimate of cotinine present in the body as described previously.[23,24] Nicotine intake was ascertained using a COT rapid test cassette (Craig Medical Distribution, Vista, California, USA), which is a lateral flow, rapid chromatographic immunoassay for the detection of cotinine in urine with a cut off concentration of 200ng/ml.[23] The test uses a monoclonal antibody to detect elevated levels of cotinine. The test was performed using the manufacturer's guidelines (International Biomedical Supplies). The presence of cotinine in the sample above the cutoff is indicated by the appearance of a colored line in the test region of the cassette. The sensitivity and specificity of the test are 99% and 94%, respectively.[23]

### Statistical analysis

Frequency statistics and cross tabulations were conducted in SPSS version 24. Differences in proportions of self-reported and cotinine verified smoking status were calculated using McNemar’s chi-squared statistic for repeated measures. A risk ratio was calculated to examine the sex differences between self-reported non-smokers who had a positive cotinine test.

Written informed consent was obtained from all study participants prior to the start of data collection. This research was approved by the Institutional Review Board(s) at New York University Abu Dhabi (NYUAD), Sheikh Khalifa Medical City (SKMC), Zayed Military Hospital (ZMH), and NYU Langone Medical Center in New York.

## Results

**Fig 1:**
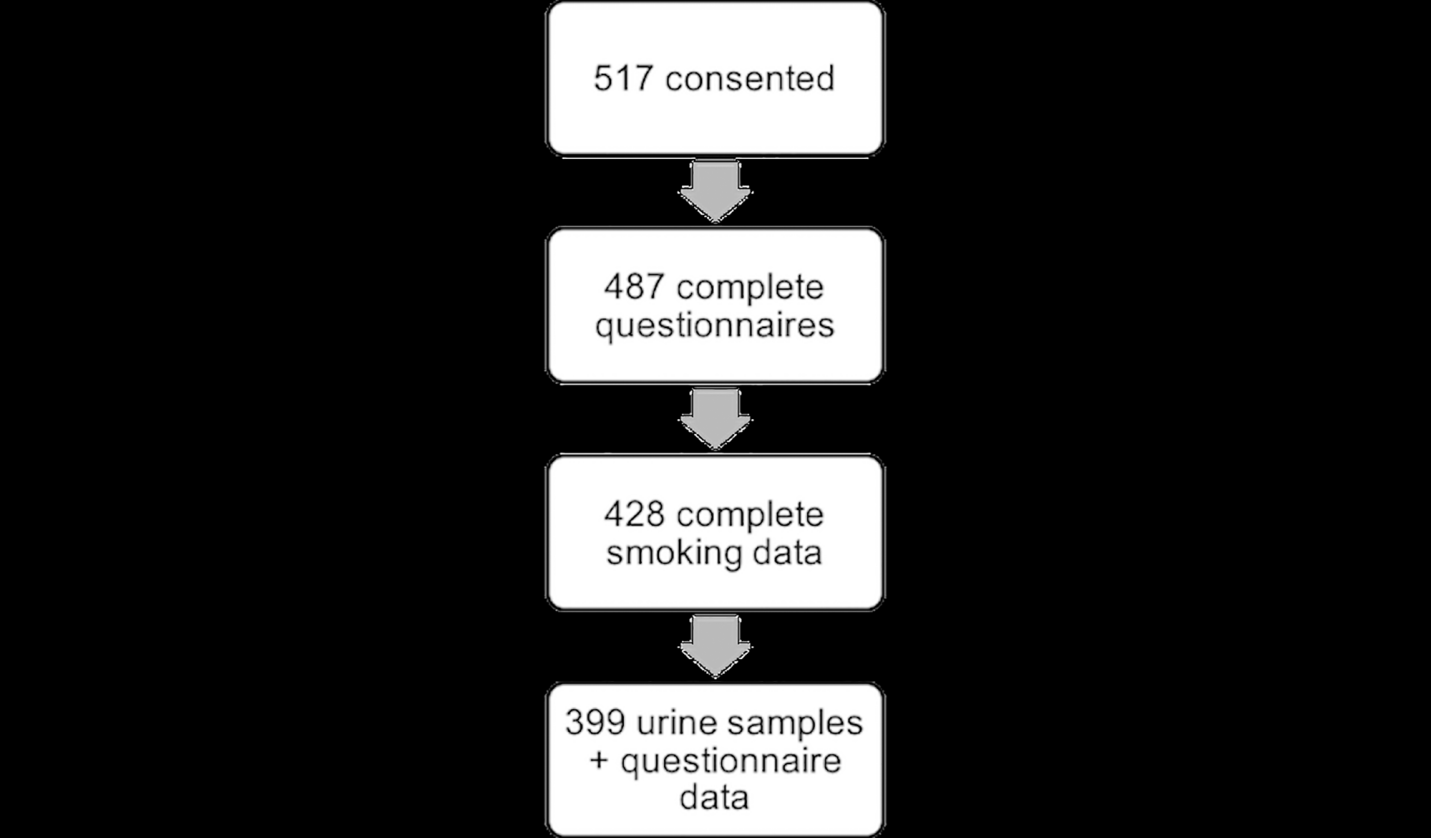
UAEHFS smoking data included for analysis.

A total of 517 participants consented to participate in the pilot study (Fig 1). Of these, 487 questionnaires were completed, with 428 containing complete data on smoking behaviors. In addition to completing a questionnaire, participants were asked to provide biological samples for laboratory testing. Urine samples were collected and cotinine values were obtained for 399 participants who also have corresponding questionnaire data.

Participant demographics are described in Table 1. Overall self-reported tobacco use was 36% among men and 3% among women. Among men, cigarettes were the most commonly used form of tobacco (23%) followed by dokha/midwakh (16%). Self-reported tobacco use among women was low, but among those who did report using tobacco, cigarettes were the most common, followed by shisha (1.9% and 1.3%, respectively). For both men and women combined, the median age for initiating smoking cigarettes was 17.5 years (interquartile range IQR = 14–19) and 20 years for midwakh (IQR = 15–25). Cigarette smokers averaged 12±8.3 cigarettes/day, with a range of 1–40 cigarettes smoked per day. Midwakh smokers averaged 10±8.5 midwakh pipes/day, with a range of 1–50 midwakh pipes smoked per day. The median age of initiation for shisha smokers was 19 years (IQR = 17–22), and they averaged smoking 4±3.2 shisha pipes/week. Approximately 30% of women and 9% of men reported some level of exposure to cigarette, midwakh or shisha smoke in the home. Most non-smokers reported they were not exposed to smoke in the home, but 17% did report some level of exposure. Table 2 summarizes the secondhand smoke exposure times for smokers and non-smokers.

**Table 1:** UAE Healthy Future Study pilot demographics.

	Male	%	Female	%	Total
	330	67.8	157	32.2	487
Age (mean±SD)	32.6±10.7		30.2±9.9		31.8±10.5
Marital status^a^					
Single	106	32.1	80	51.0	186
Married	195	59.1	56	35.7	251
Divorced	10	3.0	12	7.6	22
Widowed	0	0.0	3	1.9	3
Education^a^					
Less Primary	48	14.5	6	3.8	54
Secondary	131	39.7	69	43.9	200
≥ University	118	35.8	72	45.9	190
Other	14	4.2	4	2.5	18
Income^a^					
Less 20K	51	15.5	26	16.6	77
20-39K	78	23.6	33	21.0	111
40-59K	28	8.5	8	5.1	36
60-79K	16	4.8	4	2.5	20
80-99K	13	3.9	5	3.2	18
100K+	19	5.8	9	5.7	28
Employment^a^					
Paid/self employed	162	49.1	62	39.5	224
Working in home	41	12.4	22	14.0	63
Retired	12	3.6	1	0.6	13
Student	22	6.7	22	14.0	44
Unemployed	10	3.0	16	10.2	26
Other	64	19.4	28	17.8	92
Smoking Status^b^					
Non-smoker	213	64.5	152	96.8	365
Cigarette smoker	77	23.3	3	1.9	80
Midwakh smoker	51	15.5	1	0.6	52
Shisha smoker	41	12.4	2	1.3	43
Smoke exposure in home^b^					
None	257	77.9	98	62.4	355
Cigarette smoke	13	3.9	24	15.3	37
Midwakh smoke	16	4.8	18	11.5	34
Shisha smoke	5	1.5	11	7.0	16

^a^ Percentages may not equal 100% due to missing values,

^b^ Column totals and percentages may double count individuals who are multi-users of tobacco

**Table 2:** Secondhand smoke exposure for smokers and non-smokers in the home.

	Cigarette smoke		Midwakh smoke		Shisha smoke	
	Smoker N (%)	Non-smoker N (%)	Smoker N (%)	Non-smoker N (%)	Smoker N (%)	Non-smoker N (%)
Not exposed	116 (95)	269 (89)	112 (91)	275 (92)	119 (98)	290 (95)
1–5 hrs/wk	4 (3)	24 (8)	6 (5)	21 (7)	2 (2)	14 (5)
6–10 hrs/wk	1 (1)	5 (2)	3 (3)	2 (1)	0 (0)	0 (0)
11+ hrs/wk	1 (1)	2 (1)	1 (1)	1 (0)	0 (0)	0 (0)
Total	122	300	122	299	121	304

**Fig 2:**
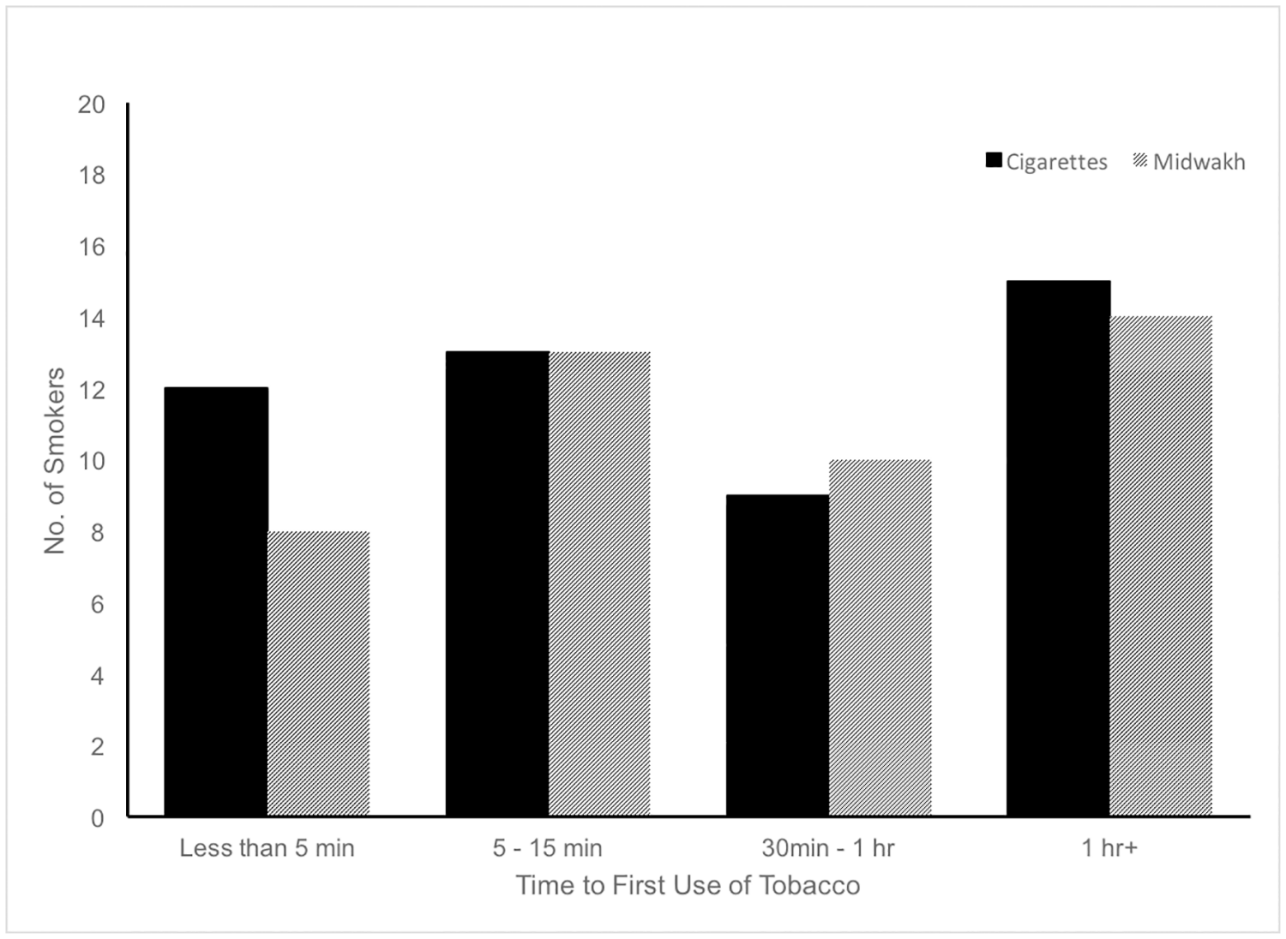
Time to first use for cigarettes and midwakh.

We assessed addiction by asking cigarette and midwakh smokers how soon after waking did they smoke and by asking shisha smokers how difficult it would be to go an entire week without smoking. Fig 2 reveals that over half of smokers who answered this question indicated that they smoked their first cigarette or midwakh within 15 minutes after waking. Approximately 75% of participants who smoked shisha reported it would be relatively easy to go an entire week without smoking shisha.

**Fig 3:**
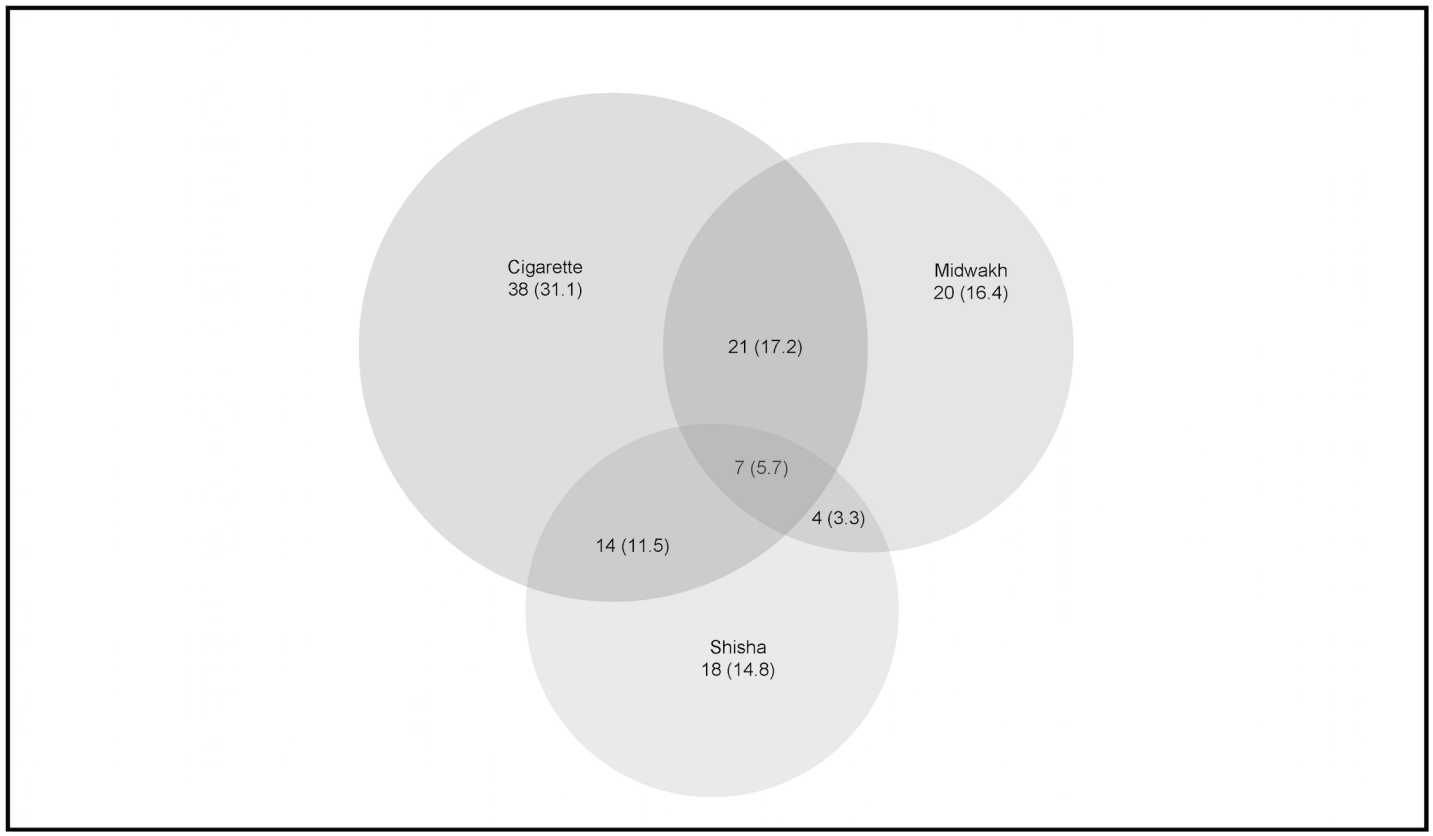
Single and poly-tobacco use by type.

Regular use of more than one tobacco product was fairly common in this study sample. Fig 3 depicts single, dual and poly-users of tobacco. Of the 122 smokers, 62% used one form of tobacco exclusively, while one-third used two forms of tobacco regularly (32%). Cigarettes and midwakh were used in combination by 17% of all smokers.

We were able to validate self-reported smoking status for 399 participants in the pilot study for whom we had urine samples and complete smoking data from the questionnaire (Table 3). The cotinine validation of smoking status was able to accurately identify 85% of people who reported current smoking. Among people who self-identified as smokers, 15% had a negative cotinine test, while nearly 14% of self-reported non-smokers tested positive for cotinine.

**Table 3:** Smoking status and cotinine validation (N = 399).

		Non-Smoker; N(%)	Smoker; N(%)
*Women*	Cot. -	103 (92)	2 (50)
	Cot +	9 (8)	2 (50)
	N	112 (100)	4 (100)
*Men*	Cot. -	150 (83)	14 (14)
	Cot +	31 (17)	88 (86)
	N	181 (100)	102 (100)
*Overall*	Cot. -	253 (86)	16 (15)
	Cot +	40 (14)	90 (85)
	N	293 (100)	106 (100)

**Fig 4:**
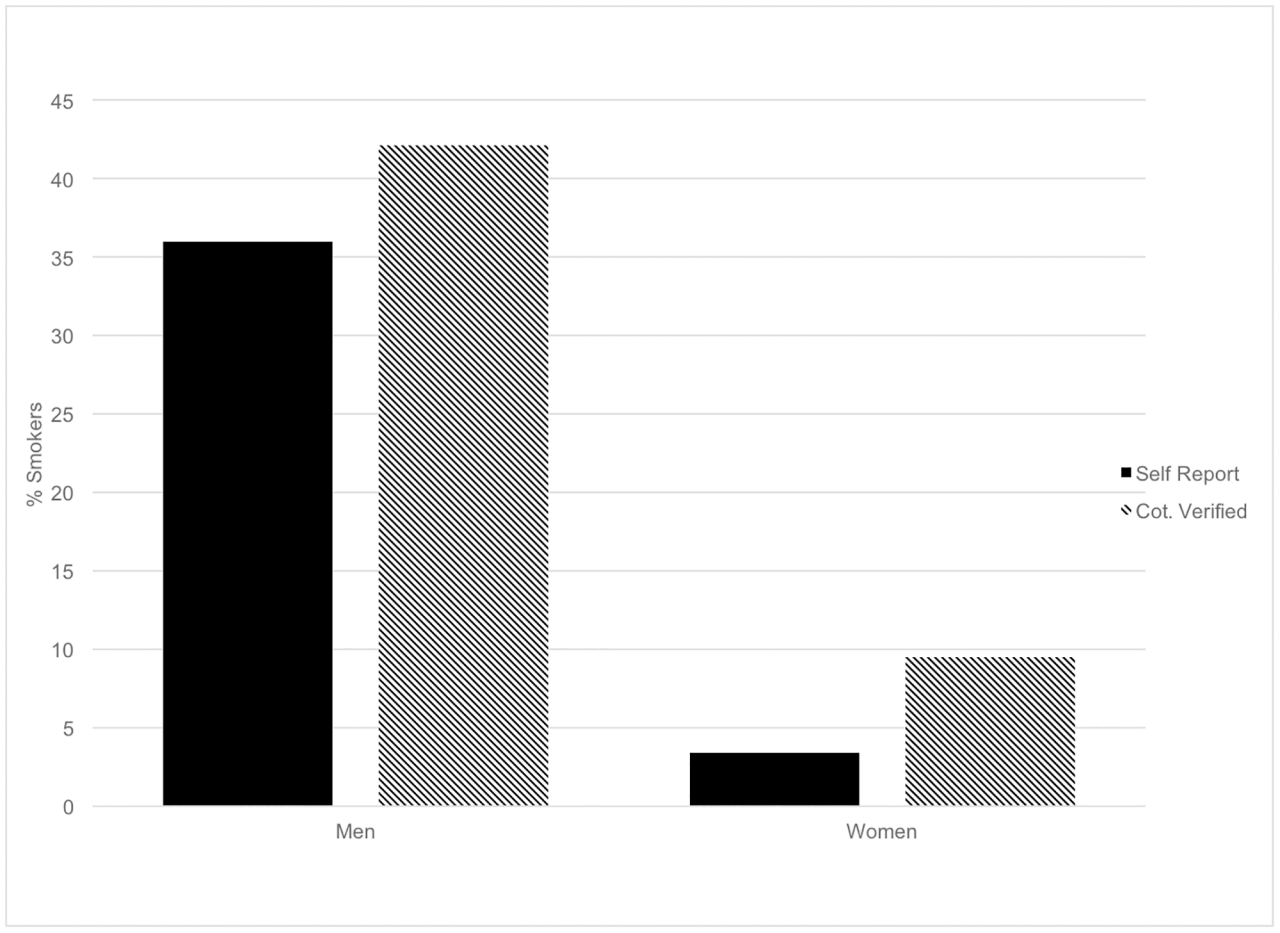
Comparison of self-reported and cotinine-verified smoking status by sex.

Among those who reported being non-smokers in Table 3, men were two times more likely to be misclassified as a non-smoker than women (17% vs. 8%). There was no difference between age groups and misclassified reporting of smoking status (OR = 1.01, 95%CI 0.56–1.82). Of the four women who self-identified as smokers, two had a negative cotinine result. Fig 4 shows the differences between self-report and cotinine verified smoking status for men and women. Self-report underestimated smoking status compared to cotinine verification among both men and women by 6 percentage points (p < 0.016 and p < 0.065, respectively).

## Discussion and limitations

Tobacco use and exposure in the Emirate of Abu Dhabi is relatively high, with 36% of men reporting tobacco use and nearly 30% of women reporting exposure to tobacco at home. Poly-use was common among men, with more than a third of men reporting dual or poly-use of tobacco. This study was the first in the region to compare cotinine levels to self-report. Based on the preliminary data from this pilot study, cotinine validation in this sample suggests that self-report of smoking status could be under reported (32% vs. 25% overall). We found rates of misclassification of smokers were higher than expected among people who reported not being a smoker. Misclassification occurred among both men and women, but was slightly higher among men in this sample.

Data from the Abu Dhabi pre-marital screening program includes information on all adult residents of the UAE applying for marriage licenses between April and December 2011. According to 2011 data from the screening program, tobacco use was estimated to be 21.6% among UAE Nationals. Among UAE National men who smoke, midwakh or dokha use (16.1%) was the most common form of tobacco followed by cigarette use (12.9%).[6] Water-pipe smoking was less common among UAE national men (5.8%) and women (0.3%).[6]

Another study of tobacco use in the UAE reported the age of onset of tobacco use varied by type. Users of cigarettes, midwakh/dokha and shisha all started in their early twenties (mean age of initiation = 22.4, 20.9, and 23.9, respectively).[15] There is evidence to suggest that use of tobacco actually begins much earlier. A study on high school students in Dubai reported that 72.1% of students have used tobacco during their lifetime.[5] Despite this study sample consisting of many nationalities, over half (54.8%) were using midwakh/dokha.[5]

Self-reported estimates of smoking among both men and women are similar to previous studies, although we found more dual and poly-tobacco use than previously reported.[6,15] Dual use of cigarettes and midwakh/dokha was the most common combination of tobacco used in this sample. Overall midwakh/dokha was found to be second most commonly used form of tobacco. Further exploration of patterns of dual and poly-tobacco use are needed.

Of all the self-identified non-smokers in the pilot, 13.7% had a positive cotinine test. Most of the misclassified non-smokers were men (17% vs. 8%). Of the four women who self-identified as smokers and had a cotinine validation test, only 2 tested positive for cotinine. The other two had undetectable levels suggesting they may have been occasional smokers. This sample size is too small to determine if there is a relationship between misclassification of smoking and other factors such as sex. We will be examining this further in the full study.

Urinary cotinine rather than cotinine in blood can be used as a marker of both active smoking and exposure to other people’s smoke due to its longer half-life and it represents exposure over days rather than hours.[24] Previous studies that have used urinary cotinine to distinguish smokers from non-smokers have used lower cut-off points and have been relatively effective in correctly classifying smokers and non-smokers.[25–27] The COT rapid test uses 200ng/ml as the cut off and provides only a positive or negative result. It does not provide information on the amount of tobacco consumption. In our study, the most likely cause of a positive cotinine test in a reported non-smoker is misclassification, that they in fact are a smoker. Sex differences in self-reporting of smoking have been found in other regions where it may be less socially acceptable for women to admit to smoking.[28] The latest Tobacco Atlas (2015) showed that most Arab countries have a high gender-based ratio in smoking. This also can be explained by the unacceptability of smoking by women within the Arab society.[29] However, it also raises the possibility of whether extensive exposure to second-hand smoke from hookahs/ waterpipe could be responsible for the positive cotinine tests in reported non-smokers. In Jordan about 82.4% of all women reported that they are exposed to cigarette smoke and 32.8% reported that they are exposed to waterpipe smoke.[30] We have previously shown that smoking hookah at home generates large amounts of airborne carbon monoxide and nicotine, even in the room adjacent to where smoking occurs.[31] Whether it is sufficient to generate a cotinine level of 200 ng/ml is unknown. Approximately 17% of non-smokers reported exposure to secondhand smoke in the home. Exposure to cigarette smoke was most common, and most non-smokers reported exposure times between 1–5 hours/week. It has been estimated that for every hour a non-smoker is exposed to secondhand smoke there is a rise of 0.45ng/mL in mean serum cotinine. [32] For future analyses, we will be collecting detailed information on second hand tobacco exposure and using a quantitative method for analyzing cotinine.

In conclusion, our study revealed overall smoking rates among Emirati nationals in Abu Dhabi were higher than previously reported. Self-reported smoking rates among women in this sample was higher than previously reported.[15] Similarly, cotinine verification revealed that women may be smoking and exposed to tobacco smoke at higher rates than previously reported.[15] Further attention should be paid to examine women’s smoking behaviors, as this has important public health implications. Additionally, further research should consider the exposure to secondhand smoke as a potential factor in examining misclassification of smoking status. Use of a test that provides more qualitative information about cotinine levels could more definitively identify non-smokers’ potential exposures to secondhand smoke. Midwakh or dokha use was the second most commonly used form of tobacco. We also found higher than expected rates of poly-tobacco use.
